# Reply to “Comment on Casiraghi et al. ‘Mucoadhesive Budesonide Formulation for the Treatment of Eosinophilic Esophagitis’ 2020, *12*, 211”

**DOI:** 10.3390/pharmaceutics12090822

**Published:** 2020-08-28

**Authors:** Antonella Casiraghi, Chiara Grazia Gennari, Umberto Maria Musazzi, Marco Aldo Ortenzi, Susanna Bordignon, Paola Minghetti

**Affiliations:** 1 Department of Pharmaceutical Sciences, Via G. Colombo 71-20133 Milan, Italy; chiara.gennari@unimi.it (C.G.G.); umberto.musazzi@unimi.it (U.M.M.); paola.minghetti@unimi.it (P.M.); 2 CRC Materiali Polimerici (LaMPo), Department of Chemistry, Università degli Studi di Milano, Via Golgi 19-20133 Milan, Italy; marco.ortenzi@unimi.it; 3 Department of Chemistry, Università degli Studi di Milano, Via Golgi 19-20133 Milan, Italy; 4 Student of Specialization School in Pharmacy, Department of Pharmaceutical Sciences, Via G. Colombo 71-20133 Milan, Italy; susanna.bordignon@gmail.com

The paper entitled “Mucoadhesive Budesonide Formulation for the Treatment of Eosinophilic Esophagitis *Pharmaceutics* 2020, *12*, 211” discusses the physicochemical and technological characterization of a formulation to treat eosinophilic esophagitis. The main critical quality attributes evaluated for each formulation were rheological properties, syringeability, mucoadhesiveness, and in vitro penetration of budesonide in porcine oesophageal tissue. These data are essential to design oesophageal delivery systems, and some of them were completely missing in previous studies. Currently, the formulation based on xanthan gum has been widely used in hospital pharmacies; nevertheless, to consider it as a gold standard, the aspects above reported required a deeper investigation. Moreover, our paper also reports the possibility to further improve the formulation and debates the addition of guar gum, which has a synergistic effect as a thickening agent.

The oral administration of budesonide to treat eosinophilic esophagitis was also reported by Hefner J et al. in “A Randomized Controlled Comparison of Esophageal Clearance Times of Oral Budesonide Preparations” [1]. In this paper, the authors concluded that *“…oral viscous budesonide slurries utilizing xanthan gum may be a superior alternative to a sucralose-based slurry due to its increased mucosal contact time and similar taste tolerance...*” This paper does not cite Zur’s article [2]. 

A similar investigation concerning the stability of this formulation was performed by Bonnet M et al. in “Formulation of a 3-months Stability Oral Viscous Budesonide Gel and Development of an Indicating Stability HPLC Method” [3]. In this paper, the authors mention that “*…previous work of Hefner and al. showed that xanthan gum had a longer esophageal mucosal contact time than sucralose. This encouraged the development of a xanthan gum-based formulation...*” These authors also proposed the same formulation without quoting Zur’s article [2].

In a meticulous analysis of the literature, we recognized the contribution of Dr Zur’s work, and we considered his work in our discussion.

Thus, even if the use of xanthan gum was earliest reported by Dr Zur, other authors mentioned above, i.e., Hefner et al. and Bonnet M et al., claimed the same conclusion.

The authors and I strongly believe that the contribution in solving the problem of the lack of adequate preparation for the treatment of a disabling childhood disease should be the main recognition and satisfaction for a researcher. Certainly, the research of many scientists can make easier the achievement of this goal. Therefore, we wish to highlight that our work presents the value of contributing to improved knowledge on this preparation, thanks to a precise characterization of the proposed formula.

## References

[1] Hefner J.N., Howard R.S., Massey R., Valencia M., Stocker D.J., Philla K.Q., Goldman M.D., Nylund C.M., Min S.B. (2016). A randomized controlled comparison of esophageal clearance times of oral budesonide preparations. Digest. Dis. Sci..

[2] Zur E. (2012). Eosinophilic esophagitis: Treatment with oral viscous budesonide. Int. J. Pharm. Compd..

[3] Bonnet M., Dermu M., Roessle C., Bellaiche M., Abarou T., Vasseur V., Benakouche S., Storme T. (2018). Formulation of a 3-months stability oral viscous budesonide gel and development of an indicating stability HPLC method. Pharm. Technol. Hosp. Pharm..

